# Design, validation, and reliability of the Bangor rugby assessment tool for evaluating technical and tactical skills in rugby union development pathways

**DOI:** 10.3389/fspor.2025.1568302

**Published:** 2025-04-23

**Authors:** George C. Lowe, Julian A. Owen, Victoria M. Gottwald, Eleri S. Jones

**Affiliations:** ^1^ Rugby Knowledge Exchange, School of Psychology and Sport Science, Bangor University, Bangor, United Kingdom; ^2^ Institute for Psychology of Elite Performance, School of Psychology and Sport Science, Bangor University, Bangor, United Kingdom; ^3^ Institute of Applied Human Physiology, School of Psychology and Sport Science, Bangor University, Bangor, United Kingdom

**Keywords:** technical, tactical, observational instrument, rugby union, talent development

## Abstract

**Introduction:**

Player profiling is fundamental to effective talent identification and development strategies. However, whilst anthropometric and physiological profiling is customary practice, effective evaluation of technical and tactical skills in team sports has arguable been overlooked, largely due to a lack of suitable measurement tools. Therefore, the aim of the present study was to design, validate, and test the reliability of a novel observational instrument for assessing technical and tactical skills in rugby union.

**Methods:**

The Bangor Rugby Assessment Tool (BRAT) was developed via the following three stages: (1) completion of a targeted literature search and expert focus group to inform initial item content; (2) Bayesian structural equation modelling (BSEM) to examine instrument factor structure; and (3) establishment of instrument reliability using intraclass correlation coefficients (ICC).

**Results:**

Results demonstrate excellent model fit (PPP = 0.511) and strong validity for both the technical and tactical factors. ICC values ranged from moderate to excellent, demonstrating good reliability (0.79).

**Discussion:**

The assessment tool offers a valid and reliable measure of technical and tactical aptitude within rugby union, whilst maintaining the requisite practical utility valued by practitioners.

## Introduction

Player profiling plays a pivotal role in effective talent identification and development systems. While anthropometric and physiological profiling is widely utilised, evaluating technical and tactical skills is equally essential (1). In rugby union and other team sports, video-based notational analysis is a commonly used method to examine key technical and tactical performance indicators (2). However, this method is constrained by issues of accessibility and cost and may be difficult to implement when assessing development of players. Alternatively, observational instruments provide researchers and practitioners with a cost-effective and accessible method to evaluate performance at regular intervals in the development process (3). Despite this, there are no observational instruments available for assessing individual players' technical and tactical skills in rugby union.

Technical indicators typically establish an athlete's level of competence to perform a particular skill, while tactical indicators refer to an athlete's “rugby IQ” via game awareness, decision making, and strategic thinking (4, 5). Whilst research supports a clear association between superior technical skill and selection/playing level (6–9), few studies have incorporated these metrics into talent identification and development research within rugby union (10). A systematic review by Dimundo et al. (10) illustrated the importance of technical and tactical skills in rugby union from a talent identification and development perspective and encourages future research to consider these factors as part of their methodologies.

As mentioned, there has been exponential growth in the use of performance analysis tools, such as notational analysis (e.g., video-based systems) and time-motion analysis (e.g., global positioning systems). While these tools provide valuable data on technical and tactical behaviours and running activities (2), in talent development, the focus remains on the technical and tactical mastery and progression of individuals. Researchers have predominantly used isolated skill tests to assess a player's technical skill, such as passing for accuracy (moving and stationary), passing for distance, kicking for distance, ground skill, and side-step ability (7, 9). However, these tests have potential positional bias and due to their detachment from the natural dynamics and game-related skills found in rugby union, may subsequently produce inaccurate predictions of a player's technical ability. From a tactical perspective, pattern recall tasks, which require players to recall structured and semi-structured tactical patterns is a commonly used assessment to assess tactical skill (11, 12). Despite this, the accessibility of this assessment limits its application in an applied sport environment. These assessments take a simplistic view of the complex and chaotic game dynamics that characterise rugby union, failing to consider the abundance of technical and tactical variables inherent in the sport. However, an observational instrument can provide coaches and researchers with an alternative assessment for the collection of multiple variables (13).

Observational instruments are commonly used field-based tools, that can facilitate the collection of detailed information on player attributes. While observational instruments have been previously used in rugby union to evaluate performance (14–16), their ability to evaluate technical and tactical skills remains unexplored. Such observational instruments would provide coaches and researchers with a cost-effective and easily accessible means with which to assess players' technical and tactical skills, serving as an alternative to video-based notational analysis and isolated skills tests. The aim of the present study was to design, validate and test the reliability of an observational instrument to assess technical and tactical skill in rugby union. The Bangor Rugby Assessment Tool (BRAT) was developed via the following three stages: (1) completion of a targeted literature search and expert focus group to inform initial item content; (2) Bayesian structural equation modelling (BSEM) to examine instrument factor structure; and (3) establishment of instrument reliability using intraclass correlation coefficients (ICC).

## Stage 1: development of the technical and tactical observational instrument

The aim of this stage was to generate a comprehensive pool of items critical for assessing technical and tactical skill in rugby union, forming the foundation for a new observational instrument. Given there are no pre-existing observational measures specifically aimed at assessing technical and tactical skill in rugby union, a targeted literature search [using a methodology similar to (17)] was carried out to identify key technical and tactical skills in rugby union. Following this, a focus group with experts was conducted to help guide the creation of the items.

### Methods

#### Participants

A purposefully selected sample of experts were invited to participate in a focus group, consisting of four coaches and one performance analyst (mean age = 36, SD = 8). The coaches were qualified at advanced (i.e., level 3) and high-performance (i.e., level 4) levels within rugby union coaching [for further detail on how coaching qualifications are positioned at the international level, see (18)]. The coaches had varying years of experience in the sport, including professional and national playing backgrounds (mean playing years = 10.33, SD = 6.43 years) and extensive coaching experience (mean coaching years = 12.75, SD = 4.57 years). Additionally, the performance analyst had experience working at the professional level and was employed by a national governing body (NGB) for rugby union. Following institutional ethical approval, all participants received an information sheet and provided written informed consent. It should be noted that written informed consent was obtained prior to participation in each stage of the study.

#### Procedure

Prior to the focus group, a draft observational instrument was created, comprising of key technical and tactical items identified through a targeted literature search. Both the literature search and focus group were conducted by the lead author, a researcher in talent identification and development who was embedded within the organisation. PubMed and Scopus databases were searched for the indicated data range. The keywords of the search included “rugby union” AND “observational instrument” OR “technical” OR “technical indicator*” OR “tactical” OR “tactical indicator*” OR “key performance indictor*” OR “game analysis” OR “performance analysis”. The inclusion criteria were as follows: included relevant data on technical-tactical indicators, notational analysis; the sport analysed was 15-a-side rugby union; involved rugby players of various genders (male and female), age (youth and adult), and playing level (regional, semi-professional, and professional); and articles were published in English. Studies were excluded if they analysed rugby league, 7-a-side rugby union, or small-sided games; were conference abstracts or doctoral theses; and did not include relevant data for the study. Articles were limited to journal articles where the full text was available. In line with Colomer et al. (19), quality of studies was not assessed based on a recognised classification method as the nature of the research valued observational, technical, and tactical studies. All articles outlined in Table 1 were evaluated for suitability by the lead author and included only if they met every item in the inclusion criteria. Additionally, guidelines provided by the NGB for rugby union, specifically key performance indicators used by performance analysts, were reviewed for further clarification on definitions and characteristics.

**Table 1:** An overview of the reviewed studies, detailing their study design, the level of competition, the number of matches included, and the performance indicators assessed.

Reference	Study design	Competition	Matches analysed	Performance indicators
Bennett et al. (20)	Quantitative study	2016–17 English Premiership Rugby Union season	127	16
Bishop and Barnes (21)	Quantitative study	2,011 Men's Rugby World Cup knockout stages	8	12
Bremner et al. (22)	Quantitative study	Two season of professional rugby union	65	19
Callinan et al. (23)	Quantitative study	Australian domestic women's rugby	47	22
Colomer et al. (24)	Qualitative analysis	Rugby World Cup		24
Colomer et al. (19)	Systematic review	International and domestic leagues	7–313	392
Cunningham et al. (25)	Quantitative study	Players from International Rugby Union squad	92	17
Hughes et al. (26)	Quantitative study	Men's 2015 and Women's 2014 Rugby World Cup knockout stages	16	25
James et al. (14)	Quantitative study	Domestic European Rugby Union team	22	16
Jones et al. (27)	Quantitative study	Domestic rugby season	20	22
Lo et al. (28)	Quantitative study	2006–16 Super Rugby	1,237	15
Mosey and Mitchell (29)	Quantitative study	2018 Queensland Premier Rugby	76	17
Ortega et al. (30)	Quantitative study	2003–06 Six Nations Championship	58	26
Ramírez-López et al. (31)	Quantitative study	2018 Under-18 Six Nations Championship	15	13
Scott et al. (32)	Quantitative study	Woman's 2017 Rugby World Cup, 2020–22 Six Nations Championship, 2019 Super Series, and 2017–22 Internation Tests	110	26
Ungureanu et al. (33)	Quantitative study	2016–17 PRO12 Championship	132	20
Ungureanu et al. (2)	Quantitative study	2018 Under-20 Six Nations Championship	5	20
Ungureanu et al. (34)	Quantitative study	2022–23 Top10 National Championship	11	17
Vaz et al. (35)	Quantitative study	International Rugby Board competitions and Super 12	324	22
Vaz et al. (36)	Quantitative study	1987–2015 Rugby World Cup finals	8	39
Watson et al. (37)	Quantitative study	2013–14 Heineken Cup, 2014–15 European Rugby Championship, 2015 Super Rugby, 2013–15 Six Nations Championship, and 2014 Rugby Championship	313	69

Following this, experts were invited to participate in a focus group. Upon arrival, the experts were briefed on the purpose of the focus group. The focus group, led by the lead author, was recorded using a Dictaphone and lasted one-hour. The experts were provided with a copy of the draft technical and tactical observational instrument and were asked to discuss the suitability for use in rugby union. Experts were asked to highlight and discuss any unsuitable items and suggest any alternative items they considered more suitable for measuring the respective technical or tactical construct. In addition to the focus group, iterative follow-up discussions with the experts were conducted to further refine the instrument. On completion of this process, the participants were de-briefed and thanked for their time. A content validity index [CVI; (38)] was used to establish which items should be retained for the next stage of the development process. A copy of the newly drafted 16-item instrument was sent to each expert with instructions to rate each item in terms of its relevance on a scale of 1–4 (1 being not relevant and 4 being highly relevant). Items that were rated as quite relevant (3) or highly relevant (4) were included in the process.

#### Statistical analyses

The focus group was transcribed verbatim into NVivo (Lumivero, Denver, US) and subsequently reviewed and analysed using Braun and Clarke (39) six-step framework for thematic analysis to identify key themes and ideas. The framework consisted of the following steps: step 1 (familiarisation), transcribing enabled researchers to immerse themselves in the data; step 2 (coding), relevant segments were coded based on item inclusion, instrument design, or notable aspects of the instruments use; step 3 (theme generation), related codes were grouped into themes reflecting technical or tactical aspect of the instrument; step 4 (theme review), preliminary themes were reviewed, modified, and developed; step 5 (theme definition), themes were further interpreted and aligned with the instrument's development; step 6 (reporting), finalised themes are presented in the discussion. Overall CVI value for each item was calculated by dividing the number of participants (*n* = 5) who rated the item as quite relevant or highly relevant by the total number of participants involved in the rating exercise process. Judgements on each item-level CVI were made as followed: >0.79 item accepted, 0.70–0.79 item revised, and <0.70 item removed (40).

### Results

**Figure 1:**
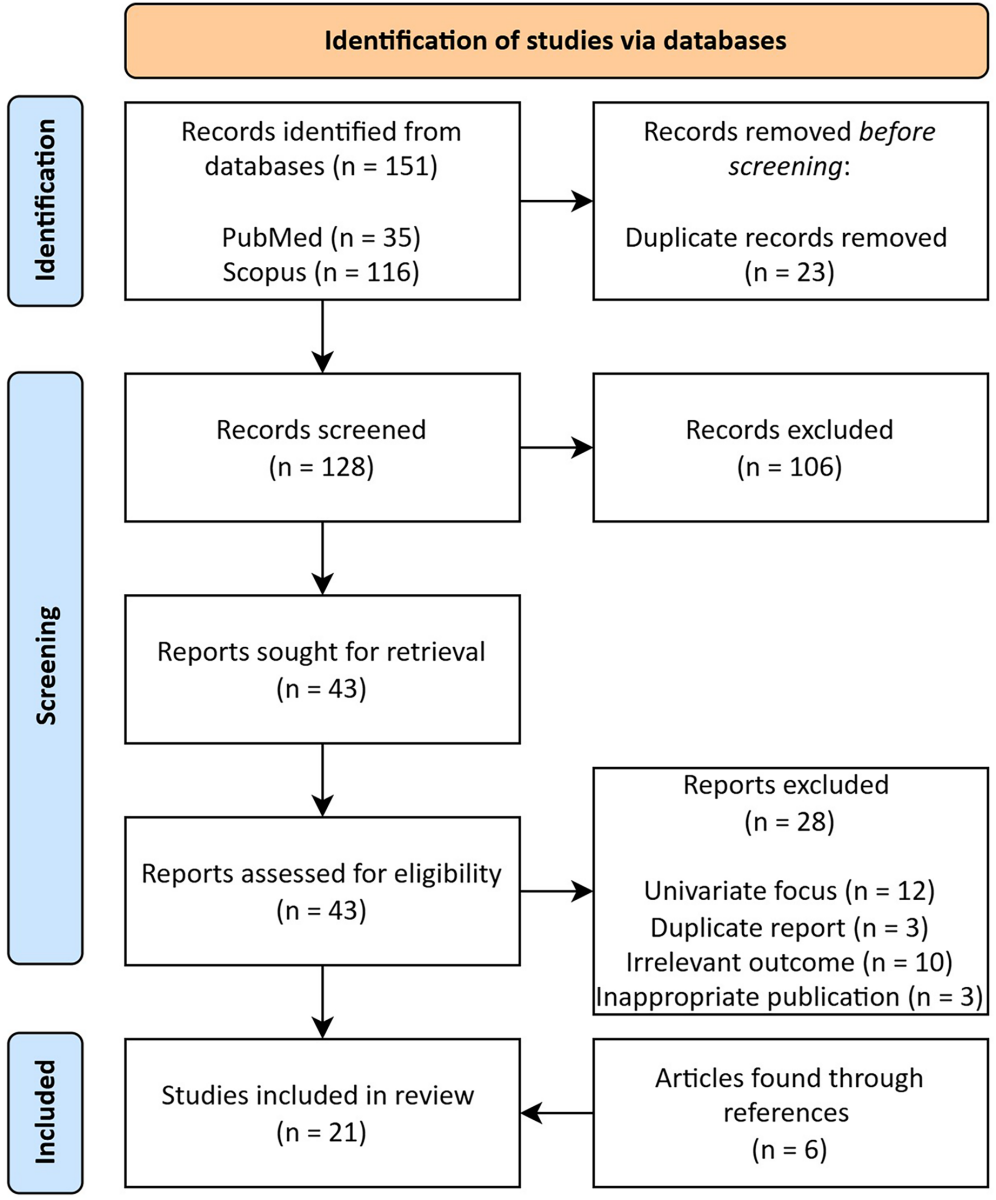
Flow diagram of the study selection process. The diagram illustrates the eligible study identification, screening, inclusion, and exclusion processes of the targeted literature search.

The aim of the targeted literature search was to identify important technical and tactical skills in rugby union. The initial search revealed 151 papers, of which 23 duplicates were removed. The remaining 128 papers were screened for eligibility based on their titles and abstracts, resulting in the exclusion of 106 papers. Forty-three papers were retained for full-text screening, of which 28 papers were excluded for reasons such as inappropriate publication (not published in a peer-reviewed journal), irrelevant outcome (study does not address technical and tactical skills), or univariate focus (study exclusively examines one skill without considering broader technical and tactical skills). In total, 21 papers met the inclusion criteria and were included in the final search. The study selection process is illustrated in Figure 1. The following variables were analysed in each study: study design, level of competition, number of events analysed, and number of technical and tactical performance indicators identified (see Table 1).

Results provided a valuable foundation for identifying important technical and tactical skills in rugby union. While the studies in Table 1 offer a detailed list of key skills, directly using these items in an observational instrument may be unsuitable due to insufficient detail in item descriptions and the extensive number of items. To address this, an initial draft of the observational instrument was developed by combining the skills identified by the literature review and NGB performance analyst guidelines. This draft instrument aimed to reduce the number of items by consolidating related skills. For example, combining skills like catching and passing into a single category, such as handling technique. Furthermore, while positional-specific skills are inherent to the sport, a decision was made to exclude these (e.g., kicking, lineout, and scrummaging). The rationale for this decision was twofold: we wanted to (1) keep the instrument global and applicable for all 15 players and positions; and (2) maintain practical utility and brevity. Following this, the draft instrument was reviewed by the focus group.

During the focus group, the participants identified several pertinent factors to consider or improve when selecting suitable items for inclusion in the newly developed BRAT. This included separating previously combined technical items such as passing, catching, and offloading, as these skills often occur independently. Furthermore, important core rugby skills (e.g., grip and ball control) and tactical items related to situational decision making (e.g., can identify and play to space, weak seams, or weak defenders) and game awareness (e.g., support play, does the player assist with attacking players who have broken through opposition defensive line) were added. In addition, greater depth of detail was emphasised on certain items. For example, participants discussed the importance of including different types of tackles in the assessment (e.g., hit, chop, and choke tackle), recognising that players execute various tackles in different game contexts.

The focus group debated and ultimately agreed that integrating pressure factors (i.e., psychological and physical stressors, as well as fatigue) adds complexity and is often hard to isolate within observational ratings. Consequently, they recognised that a simplified instrument focusing solely on technical and tactical items would be more effective and easier for observers to implement reliably and regularly. However, the overarching theme from the focus group was ensuring the instrument remains practical for coaches to use. While depth and nuance are important to reflect the complexity and numerous variables inherent in the sport, participants stressed that the instrument should remain simple enough to be applicable in the field without overcomplicating the process. Regarding the scale, the focus group discussed the use of different scales, such as 1–5, 1–7, and 1–10, as well as the potential inclusion of two separate scales to rate technical and tactical skills under pressure and without. However, the group agreed that the scale should remain academically suitable and reliable, whilst remaining detailed enough for the use to track individual progression. Consequently, a 1–7 Likert scale was agreed: 1 (below average), 2 (average), 3 (above average), 4 (good), 5 (very good), 6 (excellent), and 7 (outstanding). Following the focus group, a 16-item instrument was developed (see Table 2). CVI ratings for each item were collected. All items received scores greater than 0.79 (Table 3) and were thus accepted and retained for further examination.

**Table 2:** Bangor Rugby Assessment Tool.

Technical items
Grip and ball control (e.g., does the player hold the ball and manipulate the ball securely and effectively).
Ball carry in space (e.g., does the player carry into space).
Ball carry into contact (e.g., does the player gain ground against the opposition when entering contact or makes significant ground before recycling the ball).
Offload (e.g., does the player offload the ball effectively, at appropriate opportunities).
Catch (e.g., the player can effectively catch the ball following a pass).
Pass (e.g., the player can efficiently distribute the ball to a receiving player, who does not need to adjust their run or jump/stretch to catch the pass).
Ball presentation (e.g., player can effectively recycle and cleanly present the ball allowing for easy access and quick delivery).
Attacking contact area (e.g., does the player support the ball carrier and effectively secures the ball and the ruck, “wins the contest for possession at the ruck”).
Tackle (e.g., does the player drive the opposition backwards, stop them at the point of contact and use the appropriate type of tackle; hit, chop or choke).
Defensive contact area (e.g., does the player make any attempt to turn-over the ball through jackaling, counter rip, counter ruck, or choke attempt).
Tactical items
Consider the game situation and game plan in decision making.
Adapt quickly to transitions in play (e.g., attack to/from defence).
Can identify and play to space, weak seams, or weak defenders.
Understands and performs positional roles in attack and defence.
Urgency to reload into position.
Support play (e.g., does the player assist with attacking players who have broken through opposition defensive line).

**Table 3:** Bangor Rugby Assessment Tool I-CVI, intraclass correlation coefficients and standardised factor loading.

Item	I-CVI	Intraclass correlation coefficient (95% CI)		Standardised factor loading (95% CI)	
		Single measure	Average measure	Technical	Tactical
Grip and ball control (e.g., does the player hold the ball and manipulate the ball securely and effectively).	0.8	.64 (.40, .80)	.78 (.57, .89)	**.96** **(.90, 1.03)**	−.09 (−.19, 0)
Ball carry in space (e.g., does the player carry into space).	1	.72 (.52, .84)	.84 (.68, .92)	**.79** **(.61, .96)**	.03 (−.14, .20)
Ball carry into contact (e.g., does the player gain ground against the opposition when entering contact or makes significant ground before recycling the ball).	1	.64 (.40, .80)	.78 (.57, .89)	**.83** **(.62, 1)**	−.03 (−.20, .14)
Offload (e.g., does the player offload the ball effectively, at appropriate opportunities).	1	.54 (.04, .79)	.70 (.08, .88)	**.80** **(.61, .98)**	.00 (−.18, .18)
Catch (e.g., the player can effectively catch the ball following a pass).	1	.62 (.38, .78)	.77 (.55, .88)	**.77** **(.59, .96)**	.09 (−.10, .26)
Pass (e.g., the player can efficiently distribute the ball to a receiving player, who does not need to adjust their run or jump/stretch to catch the pass).	1	.65 (.41, .80)	.78 (.58, .89)	**.73** **(.52, .94)**	.05 (−.15, .24)
Ball presentation (e.g., player can effectively recycle and cleanly present the ball allowing for easy access and quick delivery).	1	.68 (.45, .82)	.81 (.62, .90)	**.81** **(.63, .98)**	.05 (−.13, .22)
Attacking contact area (e.g., does the player support the ball carrier and effectively secures the ball and the ruck, ‘wins the contest for possession at the ruck’).	1	.77 (.59, .87)	.87 (.74, .93)	**.80** **(.60, .97)**	.01 (−.16, .17)
Tackle (e.g., does the player drive the opposition backwards, stop them at the point of contact and use the appropriate type of tackle; hit, chop or choke).	1	.80 (.64, .89)	.89 (.78, .94)	**.73** **(.52, .92)**	.04 (−.14, .21)
Defensive contact area (e.g., does the player make any attempt to turn-over the ball through jackaling, counter rip, counter ruck, or choke attempt).	1	.74 (.55, .86)	.85 (.71, .92)	**.78** **(.55, .96)**	−.05 (−.21, .13)
Consider the game situation and game plan in decision making.	1	.54 (.10, .77)	.70 (.19, .87)	−.10 (−.20, 0)	**.95** **(.89, 1.03)**
Adapt quickly to transitions in play (e.g., attack to/from defence).	1	.71 (.51, .84)	.83 (.67, .91)	.10 (−.07, .26)	**.76** **(.59, .93)**
Can identify and play to space, weak seams, or weak defenders.	1	.71 (.49, .84)	.83 (.66, .91)	.04 (−.14, .20)	**.83** **(.67, 1.01)**
Understands and performs positional roles in attack and defence.	1	.65 (.35, .82)	.79 (.52, .90)	.06 (−.12, .22)	**.84** **(.69, 1.01)**
Urgency to reload into position.	1	.48 (.10, .72)	.65 (.19, .83)	−.13 (−.30, .05)	**.96** **(.77, 1.13)**
Support play (e.g., does the player assist with attacking players who have broken through opposition defensive line).	1	.70 (.48, .83)	.82 (.65, .91)	.06 (−.12, .23)	**.81** **(.64, .99)**

I-CVI represents the item level content validity index score. Intraclass correlation coefficients were calculated for both single and average measures with 95% confidence intervals (CI) to assess inter-observer reliability. Factor loadings and 95% CIs reflect the strength of each item on the technical and tactical latent factors. Factor loadings and 95% CIs on intended factors are in bold text.

## Stage 2: validation of the technical and tactical observational instrument

The aim of the second stage was to examine the factor structure, model fit, and rigor of the new 16-item technical and tactical observational instrument. To do so, BSEM (41) was used. This novel approach is increasingly being used in sport and exercise psychology research (42, 43), offering an alternative approach to the traditional confirmatory factor analysis using maximum-likelihood, as it acknowledges that models are likely to have small cross-loadings and co-variations across indicators.

### Methods

#### Participants

Technical and tactic data using the BRAT was collected from 294 players across four age-grades within a regional hub of a National Academy consisting of nine teams ranging from under-15 (*n* = 123; 3 teams), under-16 (*n* = 87; 2 teams), under-17 (*n* = 50; 2 teams), and under-18 (*n* = 34; 2 teams). Each team (*n* = 9) of players was rated independently by one coach, three of whom were involved in Stage 1. The coaches held coaching qualifications ranging from level 2 to level 4 [see (18)] and possessed substantial coaching experiences, with all having coached for several years within regional and national age-grade environments. The sample size (*n* = 294) was deemed sufficiently large for a model with 16 items [10 participants per item; (44)]. The flexibility of BSEM allows it to effectively model complex data structures, handling variations in age-grade by examining how scores consistently align with latent factors, such as technical and tactical skill (41). This approach enables us to validate the instrument across the developmental stages represented in our sample.

#### Measures

##### Bangor rugby assessment tool

The 16-item instrument developed in Stage 1 was utilised, assessing players' technical and tactical skill. The instrument comprises of 10 items evaluating technical skill and 6 items evaluating tactical skills, with each item rated on a 7-point Likert scale ranging from 1 (below average) to 7 (outstanding).

#### Procedure

The coaches first received training on the instrument and were then instructed to rate their respective age-grade players independently. Following the instrument's protocol, coaches were asked to “think about the player's tactical awareness and their technical ability when performing in their relevant age-grade competition”.

#### Statistical analyses

BSEM models were estimated in Mplus version 8.7. Models included noninformative priors for major loadings, and informative approximate zero cross-loading and exact zero residuals. Noninformative priors for major loadings were chosen as this is the first study to evaluate the new instrument, and no prior estimates for factor loadings were available. Consequently, prior variances for cross-loadings and residual correlations were set at N (0, .01). Indicators and factors were standardised, representing factor loadings and residual correlations with a 95% limit of ± .20, reflecting relatively small cross loadings and residual correlations (41). In line with Asparouhov and Muthén (45) and Depaoli and Van de Schoot (46) recommendations, we assessed the stability of the model by varying the prior variances, as this can influence parameter estimates. This involved re-analysing the BSEM model with smaller (.005) and larger (.015) prior variances and comparing these estimates to those obtained with a prior variance of .01. All BSEM model analysis was conducted using the Markov Chain Monte Carlo (MCMC) simulation with Gibbs sampler. Estimation was conducted using 100,000 iterations to check for convergence and stability of the estimates (41). Convergence was assessed by the potential scale reduction (PSR) test, where values between 1.0 and 1.1 indicate convergence (47). Additionally, trace plots were inspected to visually inspect the stability of means and variances across chains. All analysis on standardised data and model fit was examined by inspection of the posterior predictive *p*-value (PPP), where a PPP value around .50 is an indicator of good model fit (41).

### Results

Each parameter trace plot for the two factor, 16-item model displayed considerable overlap indicating that the parameters had converged on their posterior distribution, with autocorrelation found to be below 0.2 (48). The PSR values stayed between 1.0 and 1.1, indicating further support for adequate convergence of the model. We observed relatively smooth changes between adjacent frequency bars in the histogram, suggesting that the posterior distributions were well represented (46). The PPP estimate with prior variances specified at 0.01 was PPP = 0.511, indicating good model fit. Standardised factor loading, with 95% credibility intervals, suggested that items loaded well onto their intended factors [i.e., >.4; (49)] with insignificant cross-loading (<0.2; see Table 3). The latent factors had a strong correlation [.889 (95% CI: .830, .934), *P* < 0.001], suggesting a significant and robust relationship between technical and tactical factors. Altering prior variances from .01 to .005 and .015 did not result in any meaningful change in the convergence, parameter estimates, and fit of the model, indicating that the factor loadings and cross-loadings were stable when using larger and smaller variances.

## Stage 3: reliability of the technical and tactical observational instrument

The aim of the final stage was to assess the instrument's reliability, ensuring the instrument produces stable and repeatable results across different observers. Establishing reliability is essential for determining the utility of an instrument. In this case, inter-rater reliability is a key factor in validating observational instruments for use by multiple raters.

### Methods

#### Participants

Two observers, qualified as advanced (level 3) and high-performance (level 4) rugby coaches, independently assessed the technical and tactical skill of 37 under-17 players (forward: *n* = 17; back: *n* = 20).

#### Procedure

The coaches first received training on the 16-item BRAT instrument (from Study 2) and were then instructed to rate the players independently. Following the instruments protocol, coaches were asked to follow the procedures as outlined previously in stage 2.

#### Statistical analyses

To evaluate the inter-observer reliability of all items, an intraclass correlation coefficient (ICC) was used. ICC was selected as it quantifies both agreement and consistency between raters, making it particularly suitable for observational instruments. ICC estimates and their 95% confident intervals were calculated using IBM SPSS V.29.0.1.0 based on single (i.e., reliability from a single observer's perspective) and average (i.e., reliability when both observer's scores are combined) measures, absolute-agreement, two-way random model. ICC values were interpreted as followed: <0.5 poor reliability, 0.5–0.75 moderate reliability, 0.75–0.9 good reliability, and >0.9 excellent reliability (50).

### Results

Inter-observer reliability results from ICC analysis are reported in Table 3, including both single and average measure ICCs along with 95% confidence intervals. Across all items, the average ICC values ranged from 0.65 to 0.89, indicating moderate to good reliability (50). The single measure ICC values were generally lower but demonstrated a similar trend of moderate to good reliability, with the exception of the item “urgency to reload into position” with a value below .5 indicating poor reliability. This suggests that this item may be prone to higher variability between raters. Due to the increased susceptibility of single measure ICCs to random error and potential bias (51), we focused on the average ICC values which provide a more stable and accurate reflection of reliability across multiple raters. The average for the average measure ICC was 0.79 indicating good reliability for the instrument overall.

## Discussion

The aim of the present study was to design, validate and test the reliability of an observational instrument for assessing technical and tactical skill in rugby union. Our findings demonstrate that the observational instrument developed through a targeted literature search and focus group, shows excellent model fit (PPP = 0.511) and good reliability (ICC = 0.79). This instrument offers an alternative method to video-based notational analysis and isolated skill tests, helping researchers and practitioners address the need for more comprehensive approaches in talent identification and development research. To the best of our knowledge, this is the first study to outline the development, validation, and reliability of an observational instrument specifically for assessing technical and tactical skill in rugby union. While this study represents a crucial first step in the development, further testing is necessary as instrument development is an ongoing process.

The application of BSEM in this study allowed the developed instrument to reveal excellent model fit and factorial validity across both technical and tactical factors. The use of BSEM is becoming increasingly popular in sport and exercise psychology research (42, 43) because it overcomes limitations of traditional confirmatory factor analysis by allowing for small cross-loadings and residual correlations (52). Given the novel nature of the observational instrument and the absence of prior estimates for factor loadings, BSEM was particularly advantageous in the present study. The results provide initial support for the properties of the items within the instrument and their ability to accurately assess their respective constructs. In terms of reliability, the analysis provides support for the instrument's utility. However, it is important to acknowledge some variability in the reliability of specific items. For example, the item “urgency to reload into position” demonstrated poor reliability when evaluated by a single observer, suggesting it may be prone to higher variability between raters. This item may require further refinement to improve consistency across observers in future studies. Despite this, the instrument exhibited strong validity and good reliability across the majority of its items, making it a promising tool for evaluating technical and tactical ability in rugby union.

Previous talent identification and development research in rugby union supports technical and tactical skill as important prerequisites for player selection (53), as well as progression (54). Despite this importance, few studies have integrated these factors into their methodology (10). This may stem from a general lack of multidisciplinary approaches in this area of research (55), further compounded by the limited availability of accessible methods for researchers and practitioners to use. The present instrument, which has undergone rigorous development and validation, adds to the limited resources available to assess players' technical and tactical skill. The instrument offers significant potential to enhance talent identification and development processes in rugby union by providing a structured method for evaluating these skills, enabling coaches to systematically identify players strengths and areas of improvement. Importantly, the tool is designed for use in applied settings, allowing coaches to assess players' performance following a single game or across a series of games. To ensure fair and meaningful evaluation, it should be used when players are competing within their respective age grade competitions. Ratings should be provided post-match based on a holistic evaluation of a player's involvement across multiple phases of play, rather than being limited to isolated actions. Where resources allow, coaches may also use video analysis to support their evaluations. This approach ensures that assessments reflect game context, such as adaptability to changing scenarios and consistency in technical skill execution throughout a match. Furthermore, by focusing on overall game involvement rather than individual moments, the tool provides a more comprehensive picture of a player's technical and tactical capabilities. However, it is important to note that, while the instrument was designed to be applicable across various age-grades and playing level, it is not age-grade specific. As a result, certain items may not be relevant for specific age-grades. For example, the technical item “Offload (e.g., does the player offload the ball effectively, at appropriate opportunities)” may not be applicable for younger age-grades still developing foundational passing and catching skills, as offloading is a more advanced skill emphasised in older, more experienced players.

Beyond its contribution to talent identification processes, the instrument also has practical applications in directly supporting coaches. Informed by the focus group's emphasis on practicality, the instrument provides coaches with a valuable tool for assessing technical and tactical skills, helping to inform player development plans and monitor progression. For example, coaches can use the instrument to identify a players' strengths and areas of improvement, tailor training interventions, and track progression over time. Furthermore, the instrument's brevity and accessibility makes it a practical resource that does not require extensive time or resources. However, it is not without limitations. Future research should compare the instrument with an established measure or method for assessing technical and tactical skills to further strengthen its validity and reliability. Additionally, researchers should consider expanding the instrument to include positional-specific items, thereby increasing its practical utility for both applied and research setting.

The findings of this research provide initial support for the newly developed technical and tactical observational instrument. This study represents the initial development of the instrument, with results supporting its validity and reliability, and BSEM employed as a novel method of analysis. BRAT offers a valid and reliable means of measuring technical and tactical aptitude in rugby union, whilst maintaining the requisite practical utility valued by practitioners.

## Data Availability

The raw data supporting the conclusions of this article will be made available by the authors, without undue reservation.
